# Long non‐coding RNA NORAD promotes the occurrence and development of non‐small cell lung cancer by adsorbing MiR‐656‐3p

**DOI:** 10.1002/mgg3.2112

**Published:** 2022-11-29

Chen, T., Qin, S., Gu, Y., Pan, H. & Bian, D. “Long non‐coding RNA NORAD promotes the occurrence and development of non‐small cell lung cancer by adsorbing MiR‐656‐3p”. *Molecular Genetics & Genomic Medicine*. 2019, 7(8), e757. https://doi.org/10.1002/mgg3.757

The above article, published online on 17 June 2019 in Wiley Online Library (https://onlinelibrary.wiley.com), has been retracted by agreement between the journal Editor in Chief Dr. Suzanne Hart and John Wiley & Sons. The retraction has been agreed due to concerns raised by a third party about image manipulation. The journal team has investigated and determined that the image manipulation undermines the reliability of the data presented and the article's conclusions.

